# Frequency of EMG abnormalities in idiopathic inflammatory myopathies under the EULAR/ACR classification criteria

**DOI:** 10.1097/MD.0000000000037105

**Published:** 2024-01-26

**Authors:** Keiichi Hokkoku, Junpei Yamamoto, Yudai Uchida, Amuro Kondo, Taiji Mukai, Yuki Hatanaka, Hajime Kono, Jun Shimizu, Shunsuke Kobayashi, Masahiro Sonoo

**Affiliations:** a Department of Neurology, Teikyo University School of Medicine, Itabashi-ku, Tokyo, Japan; b Department of Internal Medicine, Teikyo University School of Medicine, Itabashi-ku, Tokyo, Japan; c Department of Physical Therapy, Tokyo University of Technology, Ota-ku, Tokyo, Japan; d Department of Neurology, Graduate School of Medicine, The University of Tokyo, Bunkyo-ku, Tokyo, Japan.

**Keywords:** amyopathic dermatomyositis, electromyography, EULAR/ACR classification criteria, hypomyopathic dermatomyositis, Idiopathic inflammatory myopathy

## Abstract

The European League Against Rheumatism/American College of Rheumatology (EULAR/ACR) classification criteria for idiopathic inflammatory myopathies (IIM) have been widely used in recent times. However, no studies have focused on electromyography (EMG) findings of IIM, considering the criteria. This study aimed to elucidate the frequency of EMG abnormalities, particularly fibrillation potentials and positive sharp waves (Fib/PSW), the most objective EMG findings of IIM. Clinical and EMG records of adult patients who were clinically diagnosed with polymyositis (PM), dermatomyositis (DM), amyopathic DM (ADM), or inclusion body myositis (IBM) were retrospectively reviewed and classified according to the EULAR/ACR classification criteria. The frequency of Fib/PSW in EMG was investigated in the recruited cases. Seventy-nine patients with clinically diagnosed IIM (44 with PM, 17 with DM, 7 with ADM, and 11 with IBM) were recruited. After classification using EULAR/ACR, 75 satisfied definite or probable IIM (61 and 14, respectively), and the frequency of Fib/PSW in this group was 95%. Furthermore, the remaining 4 patients with insufficient IIM probability also showed Fib/PSW. Fib/PSW may also be seen in cases with insufficient IIM probability not satisfying the criteria. EMG may help detect muscle involvement in these cases through Fib/PSW.

## 1. Introduction

The European League Against Rheumatism/American College of Rheumatology (EULAR/ACR) classification criteria for idiopathic inflammatory myopathies (IIM) have been widely used in recent times.^[1,2]^ The criteria comprise several essential clinical and laboratory findings but do not include electromyography (EMG) findings, as the relevant data from subjects were insufficient when the criteria were compiled.^[1]^

EMG plays an important practical role in diagnosing IIM, confirming muscle involvement in the disease.^[3–7]^ In particular, fibrillation potentials and positive sharp waves (Fib/PSW) in spontaneous EMG assessments indicate an inflammatory process that separates muscle fibers from the end plate zone, causing denervation and muscle fiber membrane instability. Several studies have revealed the association between Fib/PSW and histopathologic findings, such as segmental necrosis, fiber splitting, and regenerating fibers, typically seen in IIM.^[3,8,9]^ It is known that assessments of Fib/PSW are usually easier than voluntary assessments in myopathies, including IIM, because typical myopathic changes, such as small and short-duration motor unit potentials and rapid recruitment patterns, are indistinct in mild to moderate cases.^[3,10]^

To date, no studies have investigated the frequency of EMG abnormalities in IIM, considering the EULAR/ACR classification criteria. In this study, we aimed to elucidate the issue, focusing on the presence of Fib/PSW, which is relatively accessible and easy to evaluate in EMG.

## 2. Methods

The records of patients with a diagnosis of IIM from May 2009 to October 2021 were extracted from our laboratory database, and their clinical and EMG findings were retrospectively reviewed. Based on past clinical records, recruited IIM cases were classified using the EULAR/ACR classification criteria. Inclusion criteria were: adult patients who had a definite diagnosis of polymyositis (PM), dermatomyositis (DM), amyopathic dermatomyositis (ADM), or inclusion body myositis (IBM) based on the combination of clinical, serological, and histological findings, and the clinical course was followed up for more than 6 months; patients whose clinical records contained sufficient information to apply the EULAR/ACR classification criteria; patients whose EMG was conducted before the administration of any medication. Patients with complications from other neurological disorders were excluded.

The presence of Fib/PSW in EMG was investigated in the recruited cases. EMG was performed using disposable concentric needles of 0.45 mm in diameter and 50 mm in length (Nihon Kohden Co.) and standard EMG equipment (Neuropack, Nihon Kohden Co.). All examinations were conducted in the same EMG settings and performed by MS, our department most experienced board-certified electromyographer, or by other experienced electromyographers under the instruction of MS.

The incidence of Fib/PSW was qualitatively scored from 0 to 4 + according to our laboratory grading scale (0: No Fibs (or PSWs, same for later); 1+: Fibs (PSWs) are observed at 2 sites at least; 2+: Fibs (PSWs) are observed at half of the movements of the needle; 3+: Fibs (PSWs) are observed following almost every movement of the needle; 4+: Baseline is not visible due to profuse Fibs (PSWs)). The higher score for Fibs or PSWs was adopted in an examined muscle, and a score of 1 or higher was regarded as abnormal.

This study was approved by the Ethics Committee of the Teikyo University School of Medicine (approval number: 19-171).

### 2.1. Statistical analysis

Fisher exact test or McNemar test was used when comparing categorical data, and *P* values <.05 were considered statistically significant. All values are reported as the mean and standard error of the mean.

## 3. Results

Seventy-nine clinically diagnosed IIM patients were enrolled (35 men and 44 women, age: 64.4 ± 14.4, IIM subtype: 44 PM, 17 DM, 7 ADM, and 11 IBM). Of the 79 patients, 75 corresponded to definite or probable IIM based on the probability calculated from the variables defined by the EULAR/ACR classification criteria.^[1]^ In this group, probable IIM was solely observed in PM cases (14 cases), and all patients with other IIM subtypes satisfied definite IIM. The physician clinical diagnosis mostly coincided with the EULAR/ACR classification except for 6 cases. Two clinically diagnosed DM cases were classified as definite PM with the EULAR/ACR classification criteria. These 2 did not show a typical skin rash of DM, but perifascicular atrophy observed in muscle biopsies led to the clinical diagnosis of DM, and we classified them into the dissociated group. IIM probability did not reach possible IIM in 4 clinically diagnosed PM patients, and we classified them into the unsatisfied group. The details of recruited cases are described in Table 1. Regarding the unsatisfied group, all 4 patients showed absent or slight muscle weakness that reduced IIM probability. They were all referred to our department to investigate muscle enzyme elevation complicated by other collagen diseases, interstitial pneumonia, or oral statin therapy, although no particular muscle symptoms were present. Their diagnoses were made by the combination of EMG, serological tests, and muscle biopsy (Table 2).

**Table 1:** Summary of recruited cases.

EULAR/ACR classification	PM	DM	ADM	IBM	Dissociated*	Unsatisfied†	Total
Number of patients	40	15	7	11	2	4	79
Definite	26	15	7	11	2	0	61
Probable	14	0	0	0	0	0	14
Possible	0	0	0	0	0	0	0
Probability	90.6 ± 6.7%	99.8 ± 0.6%	98.1 ± 1.6%	97.1 ± 3.4%	99.5 ± 0.7%	33.8 ± 18.6%	
Muscle enzyme elevation (n)	93% (37/40)	80% (12/15)	57% (4/7)	73% (8/11)	50% (1/2)	100% (4/4)	84% (66/79)
Positive biopsy (n)	100% (27/27)‡	100% (3/3)	100% (1/1)	100% (9/9)	100% (2/2)	100% (2/2)	100% (44/44)

ADM = amyopathic dermatomyositis, DM = dermatomyositis, EULAR/ACR = European League Against Rheumatism/American College of Rheumatology, IBM = inclusion body myositis, PM = polymyositis.

* Clinical diagnosis and the EULAR/ACR classification were dissociated. While 2 cases were clinically diagnosed as DM, they were classified as PM according to the EULAR/ACR classification criteria.

† Unsatisfied: the cases with IIM probability <50%.

‡ Immune-mediated necrotizing myopathy, nonspecific myositis, and polymyositis were 11, 12, and 4 cases, respectively.

**Table 2:** Unsatisfied cases.

Case	1	2	3	4
Age, sex	80, M	72, M	73, M	54, F
IIM probability (%)	23	23	49	13
Clinical diagnosis	PM	PM	PM	PM
Reason for the first visit	Muscle enzyme elevation	Muscle enzyme elevation	Muscle enzyme elevation	Muscle enzyme elevation a
Subjective symptoms	Fatigue	Fatigue	Fatigue, arthralgia	Myalgia
Muscle weakness	(−)	(−)	neck flexor weakness	(−)
CK value (IU/L)	2954	803	1304	1252
Biopsy	IMNM	nonspecific myositis	N.D.	N.D.
Positive serum antibody	anti-SRP	anti-CCP	anti-ARS, anti-SSA, SSB	anti-HMGCR
Interstitial pneumonia	(+)	(+)	(+)	(−)
Other autoimmune diseases	(−)	RA	SjS	(−)

ARS = Anti-aminoacyl tRNA synthetase, CCP = cyclic citrullinated peptide, CK = creatinine kinase, HMGCR = 3-hydroxy-3-methylglutaryl-coenzyme A reductase, IIM = idiopathic inflammatory myopathy, IMNM = immune-mediated necrotizing myopathy, N.D. = not done, PM = polymyositis, RA = rheumatic arthritis, SjS = Sjögren’s syndrome, SRP = signal recognition particle.

Table 3 summarizes the Fib/PSW frequency in each IIM subtype classified by the EULAR/ACR classification criteria. Overall, Fib/PSW were almost constantly observed in the definite or probable IIM group (71 out of 75 cases: 95%) and in all 4 patients in the unsatisfied group. Examined muscles varied among IIM subtypes and patients. The biceps brachii (BB), iliopsoas (IP), and deltoid muscles were mostly examined in PM, DM, and ADM, and the BB and flexor digitorum profundus (FDP) were examined in IBM. Regarding other muscles, the number of tests was too few for further analysis. The BB EMG generally showed a high frequency of Fib/PSW (73% to 100%) in most IIM subtypes, except for ADM (0%). Among the 39 cases (22 PM, 7 DM, 7 ADM, and 3 unsatisfied cases), who underwent both the BB and IP EMGs, the latter showed higher sensitivity than the former (McNemar test, *P* = .04).

**Table 3:** Frequency of Fib/PSW.

EULAR/ACR classification	PM	DM	ADM	IBM	Dissociated	Unsatisfied
BB	89% (33/37)	73% (8/11)	0% (0/7)	100% (11/11)	100% (2/2)	75% (3/4)
Del	71% (10/14)	57% (4/7)	N. A.	N. A.	N. A.	N.A.
IP	100% (25/25)	91% (10/11)	57% (4/7)	N. A.	N.A.	100% (3/3)
FDP	N. A.	N. A.	N. A.	100% (11/11)	N. A.	N. A.
Total			95% (71/75)			100% (4/4)

ADM = amyopathic dermatomyositis, BB = biceps brachii muscle, Del = deltoid muscle; DM = dermatomyositis, EULAR/ACR = European League Against Rheumatism/American College of Rheumatology, FDP = flexor digitorum profundus, IBM = inclusion body myositis, IP = iliopsoas muscle, N.A. = not assessed, PM = polymyositis.

Regarding the ADM group, 5 out of 7 cases were further classified as hypomyopathic DM (HDM) based on the muscle involvement confirmed by the IP EMG, elevated serum muscle enzymes, or biopsy (Table 4). In one case (case 7), the IP EMG alone confirmed muscle involvement and the patient was classified as HDM.

**Table 4:** Summary of ADM cases.

Case	Age/sex	Muscle-specific antibodies	Elevated muscle enzymes	Fib/PSW in the BB	Fib/PSW in the IP	Biopsy	Subclassification
1	22/M	MDA-5	(+)	(−)	(+)	(+)*	HDM
2	69/F	MDA-5	(+)	(−)	(+)	N.D.	HDM
3	63/F	MDA-5	(−)	(−)	(−)	N.D.	ADM
4	66/M	TIF1-γ	(+)	(−)	(−)	N.D.	HDM
5	52/M	TIF1-γ	(−)	(−)	(−)	N.D.	ADM
6	47/M	Jo-1	(+)	(−)	(+)	N.D.	HDM
7	53/M	MDA-5	(−)	(−)	(+)	N.D.	HDM

ADM = amyopathic dermatomyositis, BB = biceps brachii, Fib/PSW = fibrillation potentials and positive sharp waves, HDM = hypomyopathic dermatomyositis, IP = iliopsoas, N.D. = not done.

* Perifascicular atrophy.

Among the 79 whole IIM cases, including the unsatisfied group, Fib/PSW were observed in 75 (95%), while elevated serum muscle enzymes (any of creatine kinase, lactate dehydrogenase, aspartate aminotransferase, or alanine aminotransferase) were observed in 66 cases (84%). The frequency of Fib/PSW was significantly higher than that of muscle enzyme elevation in the whole IIM cases (McNemar test, *P* =* *.03). Fib/PSW was still frequently observed among the cases without muscle enzyme elevation (10 out of 13 cases, 77%).

## 4. Discussion

According to our results, Fib/PSW can be frequently seen in definite or probable IIM cases determined by the EULAR/ACR classification criteria. Furthermore, they may also be observed in IIM cases where IIM probability does not meet the criteria due to slight or absent muscle weakness. It has been reported that muscle weakness can be slight or absent in overlap syndromes and anti-HMGCR myopathy, as seen in our cases.^[11,12]^ Muscle weakness might also be absent in anti-SRP myopathy if other organ involvement, like interstitial pneumonia, is the initial manifestation of the disease.^[13]^ In these mild myopathic cases, EMG can support the diagnosis of IIM by directly confirming muscle involvement.

When performing EMG in IIM, the appropriate selection of examined muscles is essential because the yield for Fib/PSW varies among them.^[5,6]^ In the present study, the BB EMG generally showed sufficient sensitivity for Fib/PSW in most IIM subtypes, but negative results were observed in some PM, DM, ADM, and unsatisfied cases. In these cases, an additional test on the IP was beneficial for improving the sensitivity of Fib/PSW. This finding concurred with past studies reporting the IP as a highly sensitive muscle for Fib/PSW in PM and DM.^[5,6]^

To date, the EMG findings of ADM have not been clearly identified. A systematic review by Gerami et al documented that the frequency of EMG abnormalities in ADM was 64%.^[14]^ However, the case reports mentioned often presented insufficient EMG results, lacking information about what type of abnormality was found and which muscles were examined. In this regard, our results may add more detailed information to this issue, showing that the frequency of Fib/PSW was 57% in the IP and 0% in the BB. The muscle involvement in ADM enables the diagnosis of HDM, a subgroup of ADM proposed by Sontheimer.^[15]^ Based on our results, although rare, Fib/PSW in EMG could be the sole sign of muscle involvement in ADM to be classified as HDM. Whether or not the discrimination between ADM and HDM has clinical relevance in the prognosis or treatment choice is still controversial.^[14,16,17]^ Nevertheless, EMG could increase the chance of diagnosing HDM and provide greater insights into this entity.

In IBM, our results showed that both BB and FDP EMGs may have high sensitivity for detecting Fib/PSW. The FDP EMG has been reported to be a useful test for differentiating IBM from neurogenic disorders, showing typical myopathic, that is, low-amplitude and thin motor unit action potentials.^[18]^

Muscle enzymes, including creatinine kinase, are common laboratory markers for muscle damage in IIM, but the values can be normal even in an active phase.^[19]^ Our results suggest that EMG can be a useful option for detecting muscle involvement through Fib/PSW in cases without muscle enzyme elevation.

Our study has several limitations. First, we retrospectively classified patients using the EULAR/ACR classification criteria because the criteria were not commonly used at the time of diagnosis in many cases. Hence, our study is open to information bias. However, we collected the medical information of all patients from electronic medical charts using a strictly defined protocol and believe this reduced the effect of the bias. Second, the evaluated muscles were limited. Nevertheless, the BB, IP, and FDP have shown a high frequency of EMG abnormalities in past studies investigating PM, DM, and IBM, while several proximal muscles, such as the quadriceps muscles, are reported to be less sensitive to Fib/PSW (38 to 66% for PM/DM), although they are accessible for EMG.^[5,6,18]^ Given the invasiveness of EMG, a limited number of tests on highly sensitive muscles will benefit patients. Third, our study only revealed the sensitivity of Fib/PSW for IIM but not its specificity due to the lack of a control group. Moreover, Fib/PSW are not findings that are unique to IIM as they can be observed in various neurogenic disorders and myopathies.^[3,10]^ Lastly, we could not include the results of myositis-specific antibody tests because not all the cases underwent a complete evaluation for the antibodies. Further studies will, therefore, be needed to establish the relationship between EMG and myositis-specific antibodies.

## Acknowledgments

The authors thank Dario Mattia Gatto, MD, and Christina Tache, MD, for their helpful comments and editing the manuscript.

## Author contributions

**Conceptualization:** Keiichi Hokkoku, Yuki Hatanaka, Shunsuke Kobayashi, Masahiro Sonoo.

**Data curation:** Keiichi Hokkoku, Junpei Yamamoto, Yudai Uchida, Amuro Kondo, Taiji Mukai, Yuki Hatanaka, Jun Shimizu, Masahiro Sonoo.

**Formal analysis:** Keiichi Hokkoku, Yudai Uchida, Amuro Kondo, Taiji Mukai, Yuki Hatanaka, Jun Shimizu, Masahiro Sonoo.

**Funding acquisition:** Masahiro Sonoo

**Investigation:** Keiichi Hokkoku, Yudai Uchida, Yuki Hatanaka, Masahiro Sonoo.

**Methodology:** Keiichi Hokkoku, Masahiro Sonoo.

**Project administration:** Keiichi Hokkoku, Masahiro Sonoo.

**Supervision:** Yuki Hatanaka, Hajime Kono, Jun Shimizu, Shunsuke Kobayashi, Masahiro Sonoo.

**Writing – original draft:** Keiichi Hokkoku.

**Writing – review & editing:** Keiichi Hokkoku, Hajime Kono, Jun Shimizu, Masahiro Sonoo.
